# Physiologically-Based Pharmacokinetic and Pharmacodynamic Modeling for the Inhibition of Acetylcholinesterase by Acotiamide, A Novel Gastroprokinetic Agent for the Treatment of Functional Dyspepsia, in Rat Stomach

**DOI:** 10.1007/s11095-015-1787-y

**Published:** 2015-09-09

**Authors:** Kazuyoshi Yoshii, Minami Iikura, Masamichi Hirayama, Ryoko Toda, Yoshihiro Kawabata

**Affiliations:** Central Research Laboratories, Zeria Pharmaceutical Co., Ltd, 2512-1 Numagami, Oshikiri, Kumagaya, Saitama 360-0111 Japan

**Keywords:** acetylcholine, acetylcholinesterase, functional dyspepsia, pharmacodynamics, pharmacokinetics

## Abstract

**Purpose:**

Acotiamide, a gastroprokinetic agent used to treat functional dyspepsia, is transported to at least two compartments in rat stomach. However, the role of these stomach compartments in pharmacokinetics and pharmacodynamics of acotiamide remains unclear. Thus, the purpose of this study was to elucidate the relationship of the blood and stomach concentration of acotiamide with its inhibitory effect on acetylcholinesterase (AChE).

**Methods:**

Concentration profiles of acotiamide and acetylcholine (ACh) were determined after intravenous administration to rats and analyzed by physiologically-based pharmacokinetic and pharmacodynamic (PBPK/PD) model containing vascular space, precursor pool and deep pool of stomach.

**Results:**

Acotiamide was eliminated from the blood and stomach in a biexponential manner. Our PBPK/PD model estimated that acotiamide concentration in the precursor pool exceeded 2 μM at approximately 2 h after administration. Acotiamide inhibited AChE activity *in vitro* with a 50% inhibitory concentration of 1.79 μM. ACh reached the maximum concentration at 2 h after administration.

**Conclusions:**

Our PBPK model well described the profile of acotiamide and ACh concentration in the stomach in the assumption that acotiamide was distributed by carrier mediated process and inhibited AChE in the precursor pool of stomach. Thus, Acotiamide in the precursor pool plays an important role for producing the pharmacological action.

## Introduction

Functional dyspepsia (FD) is a disease of subjective symptoms, including postprandial fullness, early satiation, and epigastric pain, which exhibits no organic abnormalities in the stomach [1, 2]. Although the pathophysiology of FD is not fully established, gastric dysmotility has been reported as a putative cause of FD. Gastric motility is mainly regulated by the cholinergic system, which is regulated by factors such as acetylcholine (ACh). ACh is released from presynaptic neurons into the synaptic cleft, where it then binds to ACh receptors and either affects gastric motility or is inactivated by acetylcholinesterase (AChE). This relationship suggests that gastric motility might be regulated via the inhibition of AChE activity and that AChE inhibitors might effectively treat patients with FD [3, 4].

Acotiamide was the first drug to receive approval for use in treating FD [5–7]. This compound inhibited AChE and enhanced the gastric motility like gastric accommodation reflex and gastric emptying rate after the oral administration of acotiamide to FD patients [8]. On the other hand some experiments were performed after the subcutaneous administration of acotiamide to rats to investigate the enhancement of gastric motility by acotiamide in detail because the exposure after the subcutaneous administration of acotiamide is higher than that after the oral administration of acotiamide in rats [9]. These experiments suggested that acotiamide in the stomach tissue might be effective for enhancement of gastric motility as the stomach tissue concentration of acotiamide was higher than the IC_50_ for AChE after the subcutaneous administration of acotiamide to rats [9]. Since the stomach tissue concentration of acotiamide could be important for the pharmacological action of acotiamide, the distribution of acotiamide to the stomach was evaluated in rats [10]. In that study, acotiamide was highly distributed into the stomach after the intravenous administration of acotiamide to rats [10]. Moreover, the integration plot analysis indicated that acotiamide was uptaken by at least two stomach compartments, which rapidly and slowly equilibrated with the blood [10]. However, as the role of these stomach compartments were unsettled, the relationship of the blood and stomach concentration of acotiamide with the pharmacological action remains unclear.

The purpose of this study was to elucidate the relationship of the blood and stomach concentration of acotiamide with the pharmacological action. For the purpose, we developed a physiologically-based pharmacokinetic and pharmacodynamic (PBPK/PD) model to describe the profiles for concentration of acotiamide and ACh in rats. In this model, the stomach compartments rapidly and slowly equilibrated with the blood were assumed as vascular space and extravascular compartment in rat stomach, respectively. In addition, ACh was chosen as the marker for the pharmacological action of acotiamide because the reported index like the motility index might be improper for the describing the time course change for the pharmacological action of acotiamide. The profiles for blood and stomach concentration of acotiamide were determined to characterize the pharmacokinetic parameters. Moreover, to confirm the elevation of ACh derived from the inhibition of AChE by acotiamide, the inhibitory effect of acotiamide on AChE *in vitro* and the increase of ACh in the stomach were determined.

## Materials and Methods

### Chemicals

**Fig. 1:**
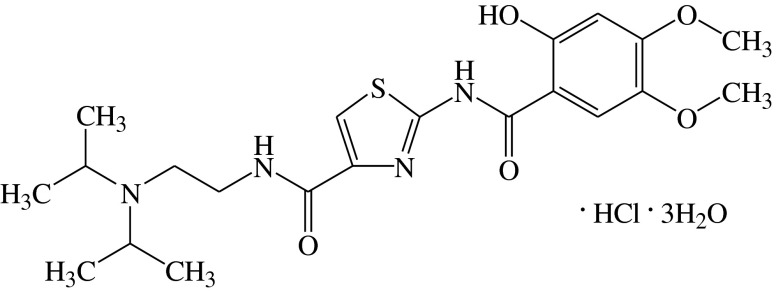
Structure of acotiamide hydrochloride.

N-[2-[bis(1-methylethyl)amino]ethyl]-2-[(2-hydroxy-4,5-dimethoxybenzoyl)amino]thiazole-4-carboxamide monohydrochloride trihydrate (acotiamide hydrochloride, Z-338/YM443, Fig. 1) and N-[2-(diisopropylamino)ethyl]-2-[(2,4,5-trimethoxybenzoyl)amino]-1,3-thiazole-4-carboxamide (ID951551, an internal standard for the determination of acotiamide) were synthesized in the central research laboratories of Zeria Pharmaceutical Co., Ltd. (Saitama, Japan). 1,1-Dimethyl-4-acetylthiomethylpiperidinium iodide (MATP+) was purchased from Dojindo Laboratories (Kumamoto, Japan). 5,5′-Dithiobis-2-nitrobenzoic acid (DTNB), and (±)-sulpiride (an internal standard for the determination of MATP + −TNB) were purchased from Sigma-Aldrich Co. LLC (St Louis, MO, USA). Acetylcholine iodide and N-ethylmaleimide (NEM) were purchased from Wako Pure Chemical Industries (Osaka, Japan). Acetylcholine-d9 chloride (an internal standard for the determination of ACh) was purchased from Toronto Research Chemicals Inc. (Ontario, Canada). All other chemicals were reagent grade.

### Animals

Male Sprague–Dawley rats (aged 6 to 7 weeks) obtained from Charles River Japan, Inc. (Kanagawa, Japan) were housed under standard controlled environmental conditions at 23 ± 3°C and 55 ± 20% humidity, with a 12-h light/dark cycle (7:00–19:00, light) and food (CRF-1; Oriental Yeast Co., Ltd., Tokyo, Japan) and water available *ad libitum*. Rats were allowed to acclimate to laboratory conditions for at least 1 week prior to performing the experiments. All animal experiments were approved by the Animal Care and Use Committee of the Central Research Laboratories of Zeria Pharmaceutical Co., Ltd.

### Preparation of Stomach Homogenate for *In Vitro* Study

Rats were anesthetized with isoflurane and their stomachs immediately excised after blood removal, rinsed with distilled water, weighed, and homogenized in distilled water to form a 50% (w/v) homogenate. Protein concentrations were determined using the Bio-Rad protein assay kit (Bio-Rad, Hercules, CA, USA).

### AChE Activity by Stomach Homogenate

AChE activity was measured using MATP+ as a substrate (50 μM), as previously described [11]. Normally, MATP+ is hydrolyzed by AChE and reacted with DTNB. The release of TNB is generally measured with a spectrophotometer at 412 nm to determine AChE activity. However, in the present study, background absorbance was extremely high in stomach homogenate (50% [w/v]), which compromised the results. We therefore measured the concentration of the MATP+ and TNB complex (MATP + −TNB), which was the result of MATP+ hydrolysis by AChE, using ultra-performance liquid chromatography (UPLC; Waters, Milford, MA, USA).

The reaction medium (0.1 M Na_2_HPO_4_ and 0.1 M NaH_2_PO_4_ including 0.5% Triton X-100, pH8.0) contained 10 mM DTNB and 2 mM NEM. Hydrolysis of MATP+ by AChE was initiated by adding 50 μM MATP+ to an incubation medium that included the stomach homogenate after preincubation at 30°C for 3 min. At designated times, 1 ml of methanol containing 0.1% formic acid was added to terminate the reaction, and an internal standard ([±]-sulpiride) was then added. Following centrifugation, the supernatant was evaporated until dry under nitrogen gas in a water bath at 60°C and the residue was dissolved in 200 μl of mobile phase B (see below for composition). The concentration of MATP + −TNB in each sample was determined by UPLC using a hydrophilic interaction chromatography (HILIC) column (ACQUITY UPLC BEH HILIC; Waters) with MATP+ as the standard. Ultraviolet absorbance was monitored at 230 nm. Mobile phase A consisted of methanol and 5 mM ammonium acetate (pH5.0) (50:50). Mobile phase B consisted of acetonitrile, isopropanol and 5 mM ammonium acetate (pH5.0) (90:5:5). A steady gradient started at 1% mobile phase A for 5 min at a flow rate of 1.0 ml/min, moving then to 50% mobile phase B over the next 4 min, and finally to 1% mobile phase A from 9 to 12 min.

The initial hydrolysis velocity was calculated using linear regression of the data taken at 60 s. The effect of acotiamide on the MATP+ hydrolysis was studied at acotiamide concentrations ranging from 0.05 to 25 μM.

### *In Vivo* Study

For the intravenous administration of acotiamide, rats were first anesthetized with isoflurane. Acotiamide was dissolved in a 5% glucose solution and then injected to 66 rats in six different experiments via the femoral vein at 1.85 μmol/kg. Blood samples were collected from the abdominal aorta at 5, 10, 15, and 30 min and 1, 2, 4, 6, 8, 24 and 48 h after drug administration and then centrifuged to separate the plasma. In addition, at each time point, the stomach was immediately excised, rinsed with distilled water, weighed, and homogenized in distilled water to form a 50% (w/v) homogenate. A portion of the homogenate from 5 min to 4 h was set aside for the determination of ACh, and the remainder was used for the determination of acotiamide concentration. The concentrations of acotiamide in plasma and stomach tissue and of ACh in stomach tissue were determined via liquid chromatography-mass spectrometry (LC-MS/MS) as described below.

### Quantification of Acotiamide by LC-MS/MS

For the quantification of acotiamide, 250 μl of each plasma sample was mixed with 10 μl of 50% methanol, 250 μl of 2% trichloroacetate, and an internal standard. The mixtures were applied to an Oasis HLB μElution solid-phase extraction plate (Waters), and analytes were eluted with 100 μl of methanol. Distilled water (100 μl) was added to each elute, and 10-μl aliquots were then subjected to LC-MS/MS.

For the stomach tissue, the stomach homogenate was further diluted with the same volume of distilled water to the homogenate. Thereafter, 10 μl of 50% methanol and 200 μl of 2% trichloroacetate were added to the solution. Following centrifugation, an internal standard was added to 300 μl of supernatant. The solution was applied to an Oasis HLB μElution solid-phase extraction plate, and the analytes were eluted with 40 μl methanol. Distilled water (40 μl) was added to each elute, and 20-μl aliquots were then subjected to LC-MS/MS.

LC-MS/MS analysis was performed on an API 4000 tandem mass spectrometer (ABSciex, Foster City, CA, USA) equipped with a solvent delivery system (LC-30 AD) and auto-injector (SIL-30 AC; both from Shimadzu, Kyoto, Japan) using a reverse phase column (CAPCELL PAK C_18_ MGII; Shiseido, Tokyo, Japan). Electrospray ionization was performed in the positive-ion mode. Acotiamide and an internal standard were quantified using the selected reaction-monitoring mode with a flow rate of 0.8 ml/min methanol/20 mM ammonium acetate (pH6.0) (6:4). The monitoring ions for acotiamide and the internal standard were *m/z* 451 → *m/z* 271 and *m/z* 465 → *m/z* 364, respectively.

### Quantification of ACh by LC-MS/MS

For the quantification of ACh, 20 μl of stomach homogenate sample was mixed with 100 μl of acetonitrile, and 10 μl of internal standard was then added to the resulting solution. After centrifugation for 10 min (16,000 × g), the supernatant was collected and subjected to LC-MS/MS.

LC-MS/MS analysis was performed on an API 4000 tandem mass spectrometer with a solvent delivery system (LC-30 AD) and auto-injector (SIL-30 AC) using a HILIC column (Atlantis HILIC; Waters). Electrospray ionization was performed in positive-ion mode. ACh and an internal standard were quantified using the selected reaction-monitoring mode at a flow rate of 0.3 ml/min acetonitrile/10 mM ammonium formate (pH 3.0) (65:35). The monitoring ions for ACh and the internal standard were *m/z* 146 → *m/z* 87 and *m/z* 155 → *m/z* 87, respectively.

### PBPK/PD Model

All plasma concentration data were converted to blood concentration data using Eq. 1, as follows:

1 $$ {C}_a={C}_p\cdot {R}_{bp} $$

where C_a_, C_p_, and R_bp_ denote the arterial blood concentration, plasma concentration, and blood-to-plasma concentration ratio (R_bp_ = 0.84), respectively.

The blood concentrations of acotiamide were fitted to Eq. 2, as follows:

2 $$ {C}_1=\frac{D\cdot \left(\alpha -{k}_2\right)}{V_1\cdot \left(\alpha -\beta \right)}\cdot {e}^{-\alpha \cdot t}+\frac{D\cdot \left({k}_2-\beta \right)}{V_1\cdot \left(\alpha -\beta \right)}\cdot {e}^{-\beta \cdot t} $$

**Fig. 2:**
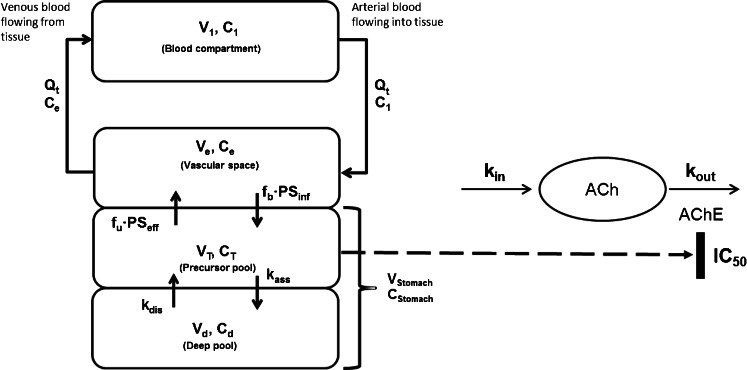
Physiologically-based pharmacokinetic and pharmacodynamic model to describe the distribution of acotiamide and AChE inhibition in rat stomach following intravenous administration. All terms and mass balance for the model are described in “Materials and Methods”.

The tissue concentrations of acotiamide were analyzed by the model shown in Fig. 2. The mass balance equations of each compartment are as follows:

Vascular space:

3 $$ Ve\cdot \frac{d{C}_e}{dt}={Q}_t\cdot \left({C}_1-{C}_e\right)-{f}_b\cdot P{S}_{\inf}\cdot {C}_e+{f}_u\cdot P{S}_{eff}\cdot {C}_T $$

Precursor pool:

4 $$ {V}_T\cdot \frac{d{C}_T}{dt}={f}_b\cdot P{S}_{\inf}\cdot {C}_e+{V}_d\cdot {C}_d\cdot {k}_{dis}-{f}_u\cdot P{S}_{eff}\cdot {C}_T-{V}_T\cdot {C}_T\cdot {k}_{ass} $$

Deep pool:

5 $$ {V}_d\cdot \frac{d{C}_d}{dt}={V}_T\cdot {C}_T\cdot {k}_{ass}-{V}_d\cdot {C}_d\cdot {k}_{ass} $$

V_d_, V_stomach_ and C_stomach_ are defined by the following equations:

6 $$ {V}_d={V}_T\cdot \frac{k_{ass}}{k_{dis}} $$

7 $$ {V}_{stomach}={V}_T+{V}_d $$

8 $$ {C}_{stomach}=\frac{A_T+{A}_d}{V_{stomach}} $$

where A_T_ and A_d_ denote the amounts of acotiamide in the precursor and deep pools, respectively; C_1_, C_e_, C_T_, and C_d_ denote the acotiamide concentrations in the arterial blood, vascular space, precursor pool, and deep pool, respectively; and V_e_, V_T_, and V_d_ denote the volumes of the vascular space, precursor pool, and deep pool, respectively. Qt and V_stomach_ represent blood flow rate and stomach tissue volume, respectively. f_b_∙PS_inf_ and f_u_∙PS_eff_ are the permeation clearances for the influx and efflux of acotiamide between the vascular space and precursor pool, respectively. The deep pool operates under linear conditions and can be characterized by first-order rate constants (k_ass_ and k_dis_). V_e_ can be described as the product of V_i_ and tissue weight [12]. Data fitting of the model equations was performed using the Phoenix model of the WinNonlin version 6.1 (Pharsight Corp., Mountain View, CA, USA).

ACh concentration in the stomach was simulated using the following model. A pharmacodynamic (PD) model was used as Model 2 [13, 14], and integrated into the PBPK model to estimate the stomach distribution of ACh. According to Model 2, the rate of change in drug response can be described as follows:

9 $$ \frac{dR}{dt}={k}_{in}-{k}_{out}\cdot \left(1-\frac{C_T}{I{C}_{50}+{C}_T}\right)\cdot R $$

where k_in_ is the zero-order constant for the production of ACh, and k_out_ defines the first-order rate constant of hydrolysis of ACh by AChE. R is the observed concentration of ACh, and IC_50_ is the acotiamide concentration that produces 50% maximal effect. Data fitting of the model equations was performed using the Phoenix model of the WinNonlin version 6.1.

## Results

### PBPK Modeling

**Fig. 3:**
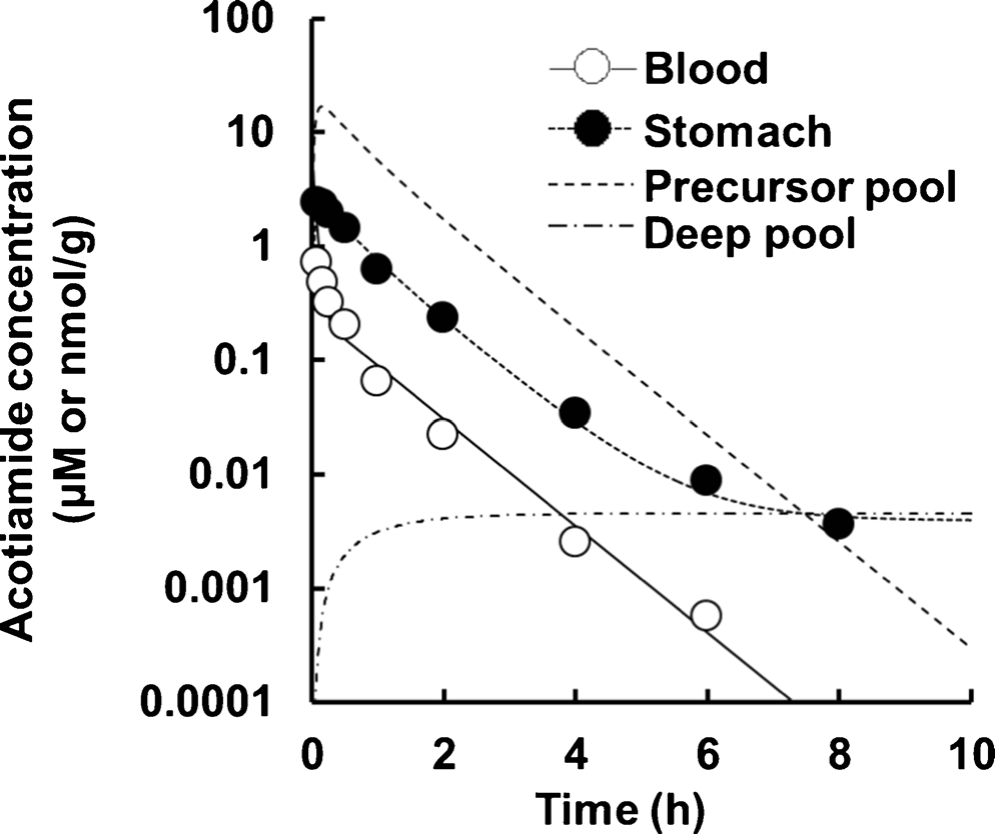
Profiles and model simulations of acotiamide concentrations in the blood, stomach, precursor pool, and deep pool after intravenous administration to rats. Each point and vertical bar represents the mean ± S.E. of six rats. All values of S.E. are inside the symbols. Open and closed circles indicate blood concentration and stomach concentration, respectively. Solid, dotted, dashed, and dashed-dotted lines represent the time course of acotiamide concentration in the blood, stomach, precursor pool, and deep pool, respectively. Symbols represent experimentally observed concentrations; lines are simulated concentration profiles using the PBPK model from Fig. 2.

Upon modeling of the disposition of acotiamide in rats, the blood and stomach concentrations of acotiamide were determined after the intravenous administration of acotiamide to rats (Fig. 3). The gastric concentration of acotiamide in the first two sampling point was approximately equal, suggesting the involvement of the time-consuming process in the distribution of acotiamide into the stomach. The assumption was supported by the result of integration plot analysis in our previous report, which showed that the distribution of acotiamide in the stomach consisted of both rapid and slow equilibrium with the blood [10]. We therefore assumed that the vascular space was involved in rapid equilibrium with the blood and the extravascular compartment was involved in slow equilibrium with the blood (Fig. 2). Moreover, the blood-flow independent membrane permeability clearance (f_b_∙PS_inf_) reported in our previous report [10], which suggested that carrier mediated uptake process was involved in the distribution of acotiamide to the stomach, was applied to the distribution process of acotiamide into the stomach. After the distribution process, acotiamide was eliminated from the blood and stomach in a biexponential manner (Fig. 3). Acotiamide in the stomach was slowly eliminated compared with the blood compartment (Fig. 3). We assumed the precursor pool where acotiamide is unbound and the deep pool where acotiamide nonspecifically interacts with cellular components (Fig. 2). Overall, acotiamide is first distributed into the vascular space, and subsequently transported from the vascular space to the precursor pool in our PBPK model.

### Analysis of Blood and Stomach Concentrations of Acotiamide

The blood and stomach concentrations were fitted to a PBPK model describing the time course of acotiamide concentration in the blood and stomach (Fig. 3). The concentration of acotiamide in the precursor pool of stomach decreased to approximately 2 μM at 2 h after administration. The concentration of acotiamide in the deep pool of stomach was estimated below 0.01 μM after the administration of acotiamide. The terminal phase for the stomach concentration was well fitted by the deep pool of stomach. The kinetic parameters for acotiamide are summarized in Table I. The fitting quality or suitability of the PBPK model was assessed using the maximized log-likelihood function (Loglik) and Akaike’s information criterion (AIC), which indicated that the developed PBPK model is appropriate for describing the kinetics of acotiamide (Fig. 3 and Table II).

**Table I:** Physiological and Pharmacokinetic Parameters for PBPK Analysis in Rats

Parameter	Value	Source
V_1_ (ml/kg)	302	By fitting
k_1_ (min^−1^)	0.126	By fitting
k_2_ (min^−1^)	0.0313	By fitting
CL_tot_ (ml/min/kg)	56.9	By fitting
V_e_ (ml)	0.441	Calculated from V_i_ [10] and tissue weight [12]
V_stomach_ (ml)	1.1	Hosseini-Yeganeh and McLachlan [12]
f_b_ · PS_inf_ (ml/min)	0.174	Yoshii *et al.*, 2011 [10]
Q_t_ (ml/min)	1.1	Hosseini-Yeganeh and McLachlan [12]
V_T_ (ml)	0.133	By fitting
f_u_ · PS_eff_ (ml/min)	0.00600	By fitting
k_ass_ (min^−1^)	0.0000320	By fitting
k_dis_ (min^−1^)	0.00000485	By fitting
IC_50_ (μM)	1.79	*In vitro* study
k_in_ (nmol/g of tissue/min)	0.00314	By fitting
k_out_ (min^−1^)	0.00415	By fitting

**Table II:** Fitting Quality of the PBPK Model for the Time Course of Acotiamide Concentrations in Blood and Stomach

Loglik	AIC
36.0	−61.9

Loglik, maximized log-likelihood function; AIC Akaike’s information criterion

Calculated using number of observations = 26 and number of parameters = 5

### Inhibition of the Hydrolysis of MATP+ by Acotiamide

**Fig. 4:**
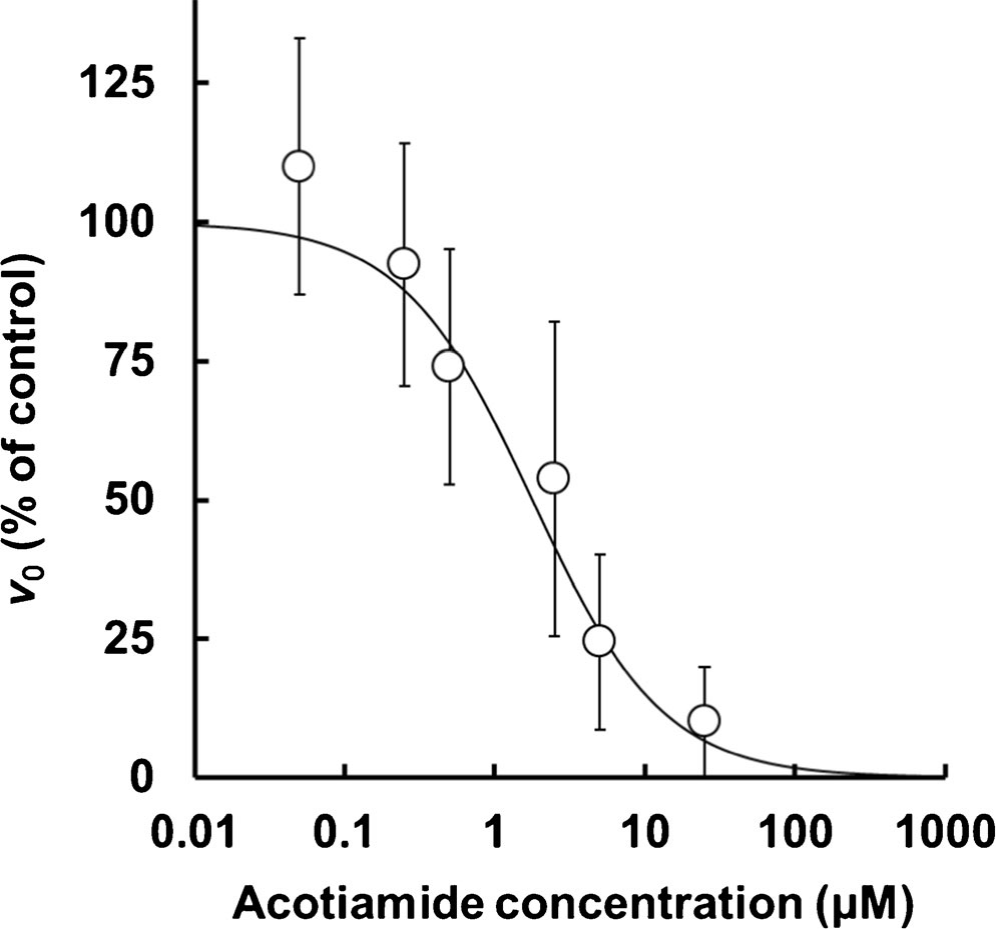
Inhibition of MATP+ hydrolysis by acotiamide. Each point and vertical bar showing the hydrolysis velocity (*v*_0_) of MATP+ represents the mean ± S.E. for three determinations.

The effect of acotiamide on AChE derived from rat stomach is shown in Fig. 4. The hydrolysis velocity of MATP+ decreased with increasing acotiamide concentration, with an IC_50_ value of 1.79 μM (Table I).

### PD Modeling

The indirect response model was linked with the PBPK model for describing the inhibition of AChE by acotiamide. The concentration of acotiamide used in the PD model was chosen as the concentration of acotiamide in the precursor pool of stomach, which covered the IC_50_ value for the inhibition of AChE by acotiamide.

### Analysis of Stomach Concentration of ACh

**Fig. 5:**
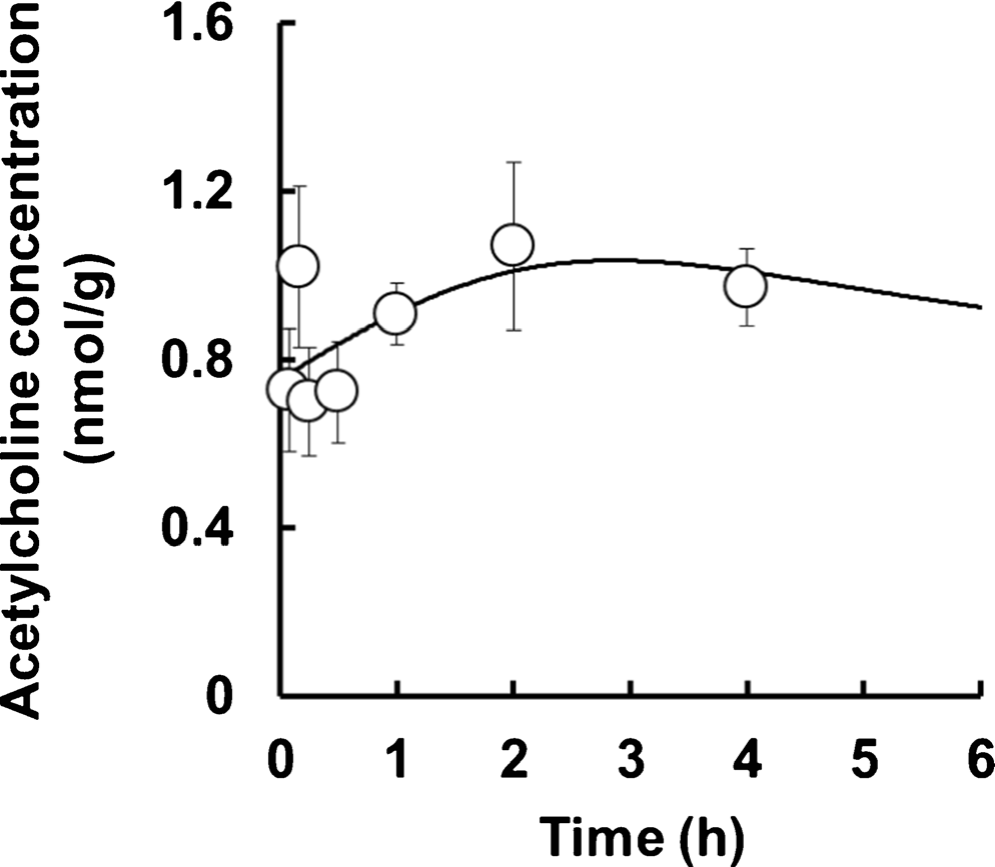
Profile and model simulation of ACh concentration in the stomach homogenate after intravenous administration of acotiamide to rats. Each point and vertical bar represents the mean ± S.E. of six rats. Symbols represent experimentally observed concentrations, while curves show the simulated concentration profiles from the PBPK/PD model (Fig. 2).

The profile of ACh after the administration of acotiamide is shown in Fig. 5. The concentration of ACh rose to 131% (% of baseline) at 2 h after administration of acotiamide. When the data of ACh concentrations was fitted to our PBPK/PD model without fixing the PD parameters (k_in_, k_out_ and IC_50_), k_in_, k_out_ and IC_50_ were 0.00337 nmol/g of tissue/min, 0.00446 min^−1^ and 2.10 μM, respectively. At that fitting, Loglik and AIC were 43.9 and −75.8, respectively, which indicated that the PBPK/PD model is appropriate for describing the kinetics of ACh. When the data of ACh concentrations was fitted to our model with fixing IC_50_ (1.79 μM), the profile of stomach concentration of ACh was well estimated by the PBPK/PD model on the assumption that acotiamide in the precursor pool of stomach inhibited AChE (Fig. 5).

## Discussion

The stomach concentration of acotiamide was higher than the blood concentration in the first time point when acotiamide concentration was measured after intravenous administration of acotiamide to rats. Acotiamide was slowly eliminated in the stomach compared to the blood. Our PBPK model well described the profile of acotiamide concentration in the blood and stomach in the assumption that acotiamide was rapidly distributed into the vascular space, uptaken to the precursor pool of stomach by carrier mediated process and permeated in the deep pool of stomach. ACh, which was determined as the marker of the involvement of acotiamide in inhibiting AChE, reached the maximum concentration in the stomach at 2 h after intravenous administration of acotiamide, while the stomach concentration of acotiamide peaked at 0.0833 h, indicating the existence of time lag between the stomach concentration of acotiamide and ACh. The unbound concentration of acotiamide in the precursor pool was higher than the *in vitro* IC_50_ value of acotiamide for inhibiting AChE activity by approximately 2 h after administration of acotiamide. The PD model well described the profile of ACh concentration in the stomach in the assumption that the unbound concentration of acotiamide in the precursor pool inhibited AChE with the *in vitro* IC_50_ for AChE inhibition. These results suggested that acotiamide inhibited AChE in the precursor pool of stomach where acotiamide was distributed by carrier mediated process.

To our knowledge, no reports have been published on a PK model for the distribution of drugs from blood to stomach as the pharmacological target although some reports have been reported on a PK model containing stomach as delivery organ of drugs to small intestine [15–17]. The precursor and deep pool of stomach were put in our model to describe the rate-limiting step in the uptake of acotiamide by stomach and slower elimination of acotiamide from stomach than blood. Acotiamide in the precursor pool of stomach plays an important role in the elevation of ACh in the stomach though the anatomical meanings of these compartments remain unclear. It’s normally difficult to estimate the microenvironmental concentration of drugs in tissue. For brain, microdialysis method was applied to estimate the unbound concentration of drugs penetrating into brain [18, 19]. The method for prediction of the unbound concentration of drugs in brain was also reported by Fridén *et al.* [20]. For stomach, the application of microdialysis to submucosa which is thicker than muscular layer and unrelated with gastric motility in the gastric layers was only reported [21, 22]. Moreover, there is no report concerning the method for prediction of the unbound concentration of drugs in stomach. Thus, we proposed a mechanism based PK model to estimate the concentration of drugs exerting their pharmacological effects in tissues like gastroprokinetic agents in stomach.

The selectivity of drug to its target is achieved by the specific affinity to drug targets, the selective access to site where drug targets exist, and/or the unique localization of drug target. For example, histamine H2 receptor, which is only involved in the regulation of gastric acid secretion among four subtypes of histamine H receptor, is a selective target for the suppressor of gastric acid secretion [23, 24]. L-type amino acid transporter 1 is consider as a specific target for antitumor because L-type amino acid transporter 1, transporting amino acids needed for the growth of cancer cells, selectively expresses in cancer cells [25, 26]. AChE, the target of acotiamide is a ubiquitous enzyme. In addition, ACh which is regulated by AChE interacts with each subtype of muscarinic receptor. There might be no selectivity on the pathway from the contact of acotiamide with AChE to the evoking of pharmacological action, so the selectivity of acotiamide to its target might be derived only from the feature in the distribution of acotiamide. Thus, it’s seems important to put in the distribution parameters to stomach in the development of our PBPK/PD model. f_b_∙PS_inf_ of the distribution parameters was calculated from the uptake clearance of acotiamide to stomach [10]. The uptake clearance can be expressed as the slope in the integration plot, where stomach to blood concentration ratio is plotted against area under the blood concentration curve to blood concentration ratio when the uptake of acotiamide was determined in a short time, during which the efflux and/or elimination of acotiamide from stomach were negligible. V_e_ of the distribution parameters (0.401 ml/g of tissue) was calculated as the product of the intercept (V_i_) of y axis of integration plot and tissue weight (V_i_ ∙ tissue weight). When V_e_ was compared with the volume of capillary bed (0.0108 ml/g of tissue) [27] and inulin space (0.295 ml/g of tissue) [10], V_e_ was similar to inulin space, an extracellular space. Therefore, f_b_∙PS_inf_ might represent the transport of acotiamide through plasma membrane of gastric cells. Moreover, the volume of precursor pool (V_T_) which was 12.1% of the volume of stomach (V_stomach_) was similar to fraction of acotiamide distributed to the stomach cytosol (12.0%) reported in our previous report [10]. The result suggested that the volume of precursor and deep pool reflected fraction of acotiamide distributed to cytosol and organelle of gastric cells, respectively.

The time lag between drug concentration and the effect was often observed [13, 14, 28]. For describing the delay of drug effect, the PD models like hypothetical effect compartment model and indirect response model were used. The hypothetical effect compartment model is useful in the case that mechanism of the delay for drug effect is unknown, while the indirect response model is available for drugs whose mechanism of the delay is derived from the mechanism of action. An indirect response model was chosen as our PD model because acotiamide is a known AChE inhibitor. Dayneka *et al.* classified the indirect response model into four models types-based on the mechanism of action [13]. Based on this classification, the effects of reversible anticholinesterase agents, which inhibit the enzymatic breakdown of ACh, demonstrated that our model could be categorized as Model 2. The rate of change in drug response using this model is described by Eq. 9, and many drugs described in this manner show a delay compared to the time course of the drug concentration. Jusko *et al.* used an indirect response model to describe the muscular response as ACh level in patients with myasthenia gravis during therapy using pyridostigmine, acetylcholinesterase inhibitor [29]. Therefore, the indirect response model is suitable for describing the inhibitory effect of acotiamide on AChE in rat stomach.

Normally, acetylthiocholine (ATCh) is used to evaluate AChE activity [30]. However, ATCh is a known substrate of both AChE and butyrylcholinesterase (BChE), meaning BChE inhibitors such as iso-OMPA need to be included in the incubation medium when investigating acotiamide effects [31]. Recently, Kikuchi *et al.* reported that MATP+ was suitable AChE selective substrates for detecting of AChE activity [11]. Therefore, in this study, we used MATP+ as substrate of AChE for investigation of the effects of acotiamide on the AChE activity. Hydrolysis velocity of MATP+ was decreased by the addition of acotiamide, with the IC_50_ value of 1.79 μM. Kawachi *et al.* have reported that acotiamide inhibited ATCh hydrolysis by stomach homogenate with IC_50_ value of 2.3 μM [9]. Furthermore, acotiamide also inhibited human AChE with Ki value of 0.61 μM [31]. Our observation was similar to these previously results, therefore, it was considered that using of MATP+ for investigation of AChE inhibition is appropriate.

In conclusion, we elucidated the relationship of the blood and stomach concentration of acotiamide with the pharmacological action. Acotiamide was distributed by carrier mediated process and inhibited AChE in the precursor pool of stomach. Acotiamide in the precursor pool plays an important role for producing the pharmacologic action. Moreover, we proposed PBPK/PD model to describe the pharmacologic action of acotiamide in stomach.

## Abbreviations

ACh: Acetylcholine
AChE: Acetylcholinesterase
FD: Functional dyspepsia
PBPK: Physiologically-based pharmacokinetic
PD: Pharmacodynamic
